# Silent giant: a 35-cm mucinous ovarian cystadenoma presenting as failure to lose weight

**DOI:** 10.1093/jscr/rjag297

**Published:** 2026-04-23

**Authors:** Eduardo A Román Cataña, Paula Castellanos, Diego Cornejo, Miguel Rueda Mesías, Santiago A Muñoz-Palomeque

**Affiliations:** Department of Surgery, Hospital Metropolitano, Av. Mariana de Jesús OE7-47, Quito, Pichincha 170517, Quito, Ecuador; Obstetrics and Gynecology Resident, PGY 1, Universidad Internacional del Ecuador-Hospital Metropolitano, Av. Mariana de Jesús OE7-47, Quito, Pichincha 170517, Ecuador; Department of Gynecology, Hospital Metropolitano, Av. Mariana de Jesús OE7-47, Quito, Pichincha 170517, Ecuador; Department of Surgery, Division of General and Oncologic Surgery, Hospital Metropolitano, Av. Mariana de Jesús OE7-47, Quito, Pichincha 170517, Ecuador; General Surgery Resident, PGY 4, Universidad Internacional del Ecuador-Hospital Metropolitano, Av. Mariana de Jesús OE7-47, Quito, Pichincha 170517, Ecuador

**Keywords:** giant ovarian cyst, mucinous cystadenoma, O-RADS, hydronephrosis, adnexal mass

## Abstract

Giant ovarian mucinous cystadenomas have become uncommon due to earlier detection, yet they may closely mimic malignancy on imaging. We report a 45-year-old woman with progressive abdominal enlargement and inability to lose weight, who remained otherwise asymptomatic. Imaging revealed a multiloculated right adnexal mass with thick septations and apparent solid components, associated with minimal ascites and right grade III hydronephrosis. Based on imaging features, the mass was classified as O-RADS 5, while serum tumor markers were within normal limits. Exploratory laparotomy demonstrated a 35-cm right ovarian mass with marked anatomical distortion and dense Zühlke grade IV adhesions. The tumor was removed intact to avoid spillage, and intraoperative frozen section analysis confirmed benign pathology, guiding continuation of the planned procedure. Final histopathology revealed a benign mucinous cystadenoma. Postoperative recovery was uneventful. This case highlights the limitations of imaging in giant ovarian tumors and underscores the importance of a safety-oriented surgical approach.

## Introduction

Mucinous ovarian cystadenomas account for ~10%–15% of benign ovarian neoplasms. [1, 2] With widespread access to imaging, tumors exceeding 30 cm have become rare [2]. However, when they reach giant proportions, their radiologic appearance may mimic borderline or malignant epithelial tumors, frequently leading to high-risk classifications under systems such as O-RADS [3, 4] We report a giant mucinous cystadenoma presenting solely as failure to lose weight, highlighting diagnostic, and surgical challenges.

## Case report

A 45-year-old woman presented with progressive abdominal enlargement and inability to lose weight. She denied abdominal pain, gastrointestinal symptoms, or urinary complaints. Her last gynecological evaluation had been performed ˃10 years prior to presentation. Past medical history included hysterectomy for uterine leiomyomatosis, bilateral salpingectomy, cesarean sections, childhood epilepsy, and migraine. Physical examination revealed a globose abdomen with a large palpable mass and a positive ascitic wave.

**Figure 1:**
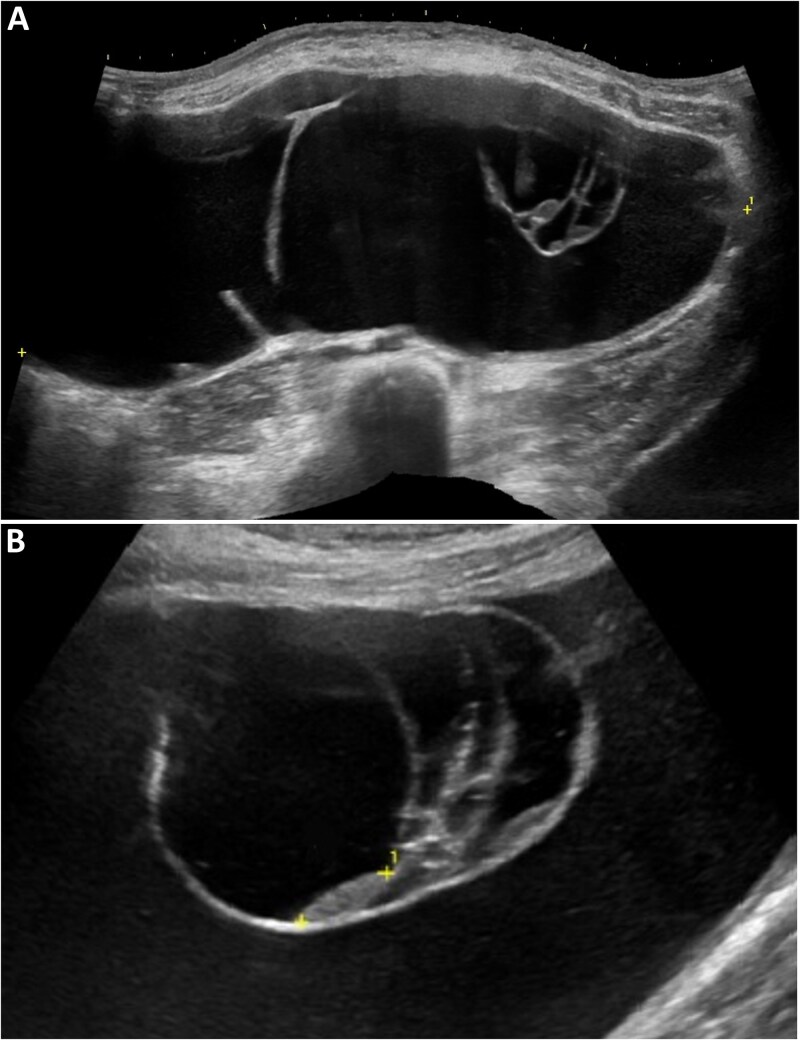
Transabdominal ultrasound. (A) Panoramic transabdominal ultrasound demonstrating a giant multiloculated cystic mass occupying the abdominopelvic cavity, with predominantly anechoic content. (B) Detailed view showing internal septations, some appearing thickened, without definite solid nodules or vascularized components.

**Figure 2:**
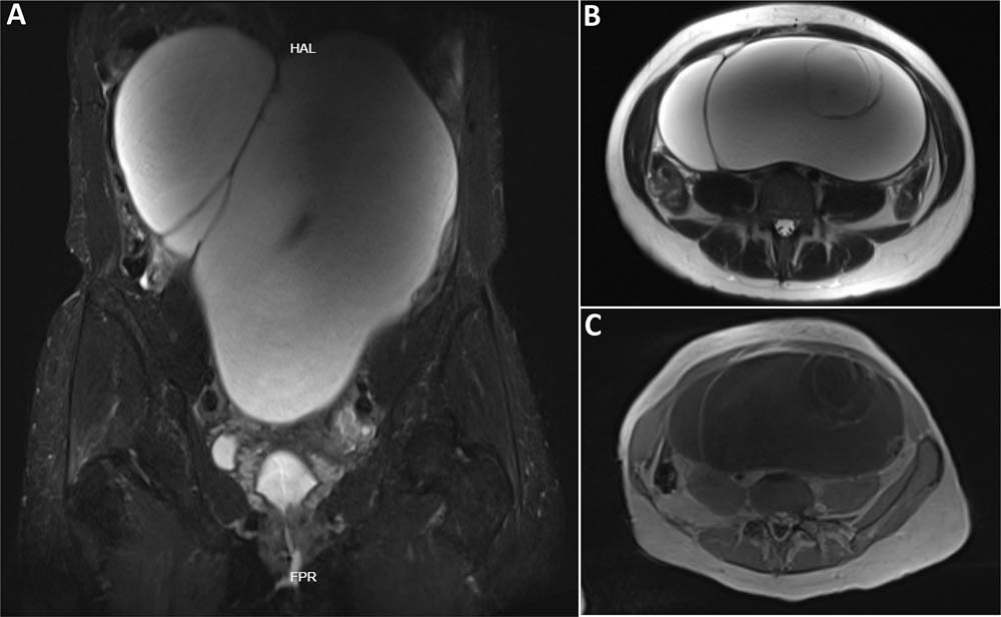
MRI of the abdominopelvic mass. (A) Coronal T2-weighted image with fat suppression (TIRM) demonstrating a giant multiloculated cystic lesion occupying most of the abdominal and pelvic cavity. (B) Axial T2-weighted (HASTE) image showing thick internal septations, consistent with a complex adnexal mass. (C) Axial T1-weighted post-contrast (VIBE-DIXON) image demonstrating enhancement of thick internal septations, findings consistent with a high-risk adnexal lesion (O-RADS MRI 5) and supporting the indication for surgical management.

Ultrasound demonstrated a multiloculated adnexal mass measuring 23 × 16 × 9 cm (Fig. 1). Magnetic resonance imaging (MRI) revealed a 25 × 20 × 15 cm multiloculated right adnexal mass with thick enhancing septations, peripheral solid-appearing components, minimal ascites, and right grade III hydronephrosis secondary to tumor-related ureteral compression. Based on imaging features the mass was classified as ORADS-5 cm (Fig. 2). Serum tumor markers (CA-125, CA 19-9, CA 15-3, AFP, CEA, and HE4) were within normal limits.

**Figure 3:**
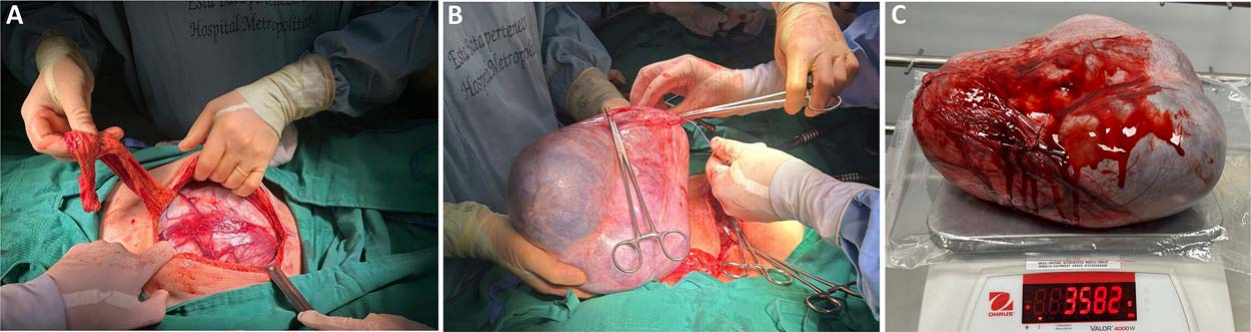
Intraoperative and gross surgical findings. (A) Intraoperative view demonstrating a giant ovarian cystic mass occupying the abdominal cavity, with intact capsule, prior to resection. (B) Gross appearance of the resected ovarian mass, removed intact, confirming complete surgical excision. (C) Gross specimen placed on a surgical scale, demonstrating a total weight of ~3.6 kg, highlighting the extreme size of the lesion.

A midline laparotomy was performed. Intraoperatively, normal anatomy was markedly distorted due to the massive size of the tumor and the presence of dense Zühlke grade IV adhesions. Careful identification of anatomical planes was undertaken, including localization of the right ureter, sigmoid colon, urinary bladder, and major vascular structures. The tumor pedicle was identified and securely clamped. Given the suspicion of malignancy, the mass was removed intact to avoid rupture and potential dissemination (Fig. 3). Intraoperative frozen section analysis revealed benign pathology, allowing continuation of the planned procedure. Dense adhesions required extensive enterolysis. Final procedures included bilateral oophorectomy, salpingectomy, and partial omentectomy. Estimated blood loss was 100 mL.

**Figure 4:**
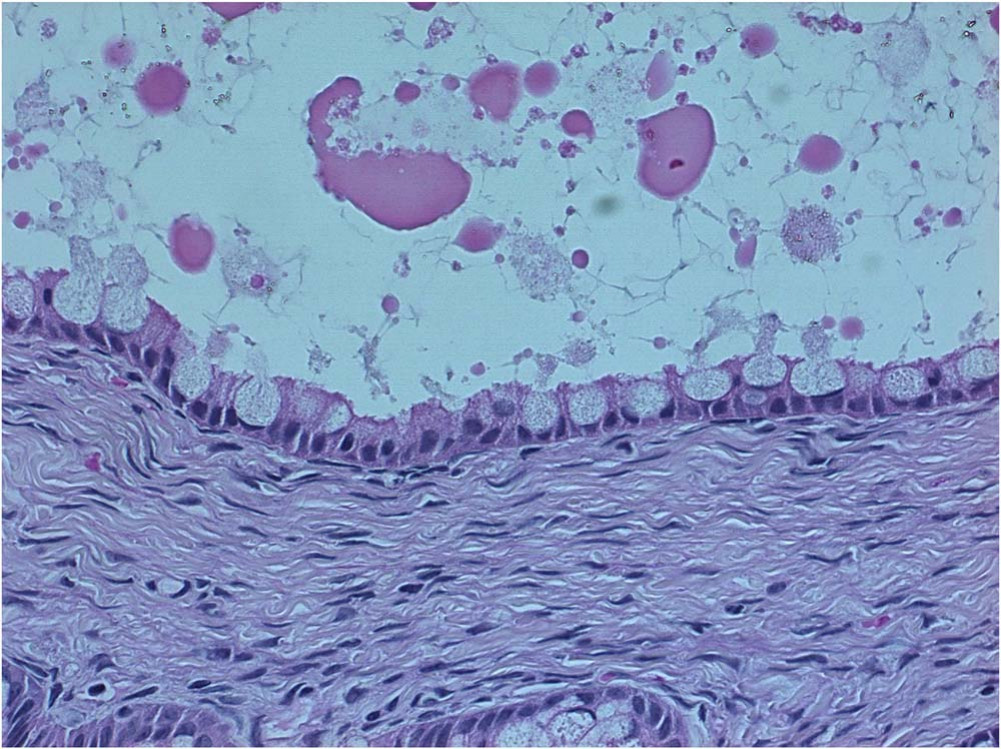
Histopathological examination of the resected ovarian mass (hematoxylin and eosin stain, ×40) demonstrating a benign mucinous ovarian cystadenoma, characterized by a single layer of mucin-secreting columnar epithelium lining the cyst wall and resting on a fibrous stroma, without cytologic atypia or stromal invasion.

Final histopathology confirmed a mucinous cystadenoma lined by single-layer mucin-secreting columnar epithelium without atypia or stromal invasion (Fig. 4). The postoperative course was uneventful, and the patient was discharged on postoperative day two. Follow-up imaging demonstrated complete resolution of the right-sided hydronephrosis, confirming its secondary nature due to tumor-related ureteral compression.

## Discussion

Giant mucinous ovarian cystadenomas remain clinically relevant despite their rarity, as they may grow silently to extreme sizes [5–7]. Slow expansion allows progressive adaptation of surrounding structures, explaining the absence of symptoms despite significant anatomical distortion and hydronephrosis [8, 9].

Radiologic evaluation of giant mucinous tumors is challenging. Features such as multilocularity, thick septations, and heterogeneous signal intensity overlap with borderline and malignant ovarian neoplasms [2, 3]. In large masses, compression of septations and distortion of anatomy may generate pseudo-solid areas, leading to high-risk ORADS classifications [10]. MRI specificity decreases significantly for adnexal masses exceeding 20–25 cm [3, 11, 12].

From a surgical perspective, giant ovarian tumors pose substantial technical challenges. In our case, marked anatomical distortion and dense Zühlke grade IV adhesions necessitated meticulous dissection and identification of critical structures. Intact removal of the mass was prioritized to minimize the risk of intraperitoneal spillage [13]. Intraoperative frozen section analysis was pivotal in guiding surgical management and avoiding unnecessary radical procedures [2, 14].

Laparotomy remains the preferred approach for giant ovarian tumors when malignancy cannot be excluded [13]. This case highlights the potential discordance between imaging-based risk stratification and final histopathology, as radiologic features consistent with a high-risk lesion (O-RADS 5) ultimately corresponded to a benign mucinous cystadenoma. Such discordance appears to be more frequent in giant ovarian masses, where size and structural complexity may overestimate malignant potential on imaging [4, 11]. Additionally, the absence of symptoms and the unusual presentation as failure to lose weight further contributed to the diagnostic challenge.

## Conclusion

Giant mucinous ovarian cystadenomas may remain clinically silent and closely mimic malignancy on imaging studies. Surgical excision is essential for definitive diagnosis and treatment. Careful intraoperative assessment, intact tumor removal, and frozen section analysis are critical for safe and effective management.

## References

[1] Mills AM, Shanes ED. Mucinous ovarian tumors. Surg Pathol Clin 2019;12:565–85. 10.1016/j.path.2019.01.008 31097115

[2] Brown J, Frumovitz M. Mucinous tumors of the ovary: current thoughts on diagnosis and management. Curr Oncol Rep 2014;16:389. 10.1007/s11912-014-0389-x 24777667 PMC4261626

[3] Lamghare P, Paidlewar S, Arkar R et al. MRI evaluation and characterization of ovarian lesions based on ovarian-adnexal reporting and data system MRI. Cureus 2024;16:e67904. 10.7759/cureus.67904 39328653 PMC11426925

[4] Byun JY . MR imaging findings of ovarian cystadenofibroma: clues for making the differential diagnosis from ovarian malignancy. Korean J Radiol 2006;7:153–5. 10.3348/kjr.2006.7.3.153 16969043 PMC2667595

[5] Akhras LN, Akhras LN, Faroog S et al. A 27-kg giant ovarian mucinous cystadenoma in a 72-year-old postmenopausal patient: a case report. Am J Case Rep 2019;20:1601–6. 10.12659/AJCR.917490 31672957 PMC6849502

[6] Alobaid A, Elamir H, Abuzaid M et al. An extremely giant ovarian mucinous cystadenoma. Gulf J Oncol 2019;1:83–6. 30956200

[7] Mishra S, Yadav M, Walawakar SJ. Giant ovarian mucinous cystadenoma complicating term pregnancy. JNMA J Nepal Med Assoc 2018;56:629–32. 10.31729/jnma.3163 30376010 PMC8997304

[8] Albers CE, Ranjit E, Sapra A et al. Clinician beware, giant ovarian cysts are elusive and rare. Cureus 2020;12:e6753. 10.7759/cureus.6753 32140321 PMC7039362

[9] Mülayim B, Gürakan H, Dagli V et al. Unaware of a giant serous cyst adenoma: a case report. Arch Gynecol Obstet 2006;273:381–3. 10.1007/s00404-005-0087-x 16249904

[10] Strachowski LM, Jha P, Phillips CH et al. O-RADS US v2022: an update from the American College of Radiology’s ovarian-adnexal reporting and data system US committee. Radiology 2023;308:e230685. 10.1148/radiol.230685 37698472

[11] Florin M, Dabi Y, Bazot M et al. How to integrate O-RADS MRI scores with clinical and imaging criteria to predict histopathological subtypes. Eur Radiol 2025;36:3017–30. 10.1007/s00330-025-11937-y 41026202

[12] Sadowski EA, Maturen KE, Rockall A et al. Ovary: MRI characterisation and O-RADS MRI. Br J Radiol 2021;94:20210157. 10.1259/bjr.20210157 33929901 PMC9327753

[13] Osawa N, Chikazawa K, Imai K et al. Oncological safety of minimally invasive surgery in borderline ovarian tumor and ovarian cancer: a retrospective comparative study. J Gynecol Oncol 2024;36:e46. 10.3802/jgo.2025.36.e46 39575999 PMC12099052

[14] Tempfer CB, Polterauer S, Bentz EK et al. Accuracy of intraoperative frozen section analysis in borderline tumors of the ovary: a retrospective analysis of 96 cases and review of the literature. Gynecol Oncol 2007;107:248–52. 10.1016/j.ygyno.2007.06.008 17631951

